# Passive Immune-Protection of *Litopenaeus vannamei* against *Vibrio harveyi* and *Vibrio parahaemolyticus* Infections with Anti-*Vibrio* Egg Yolk (IgY)-Encapsulated Feed

**DOI:** 10.3390/ijms17050723

**Published:** 2016-05-17

**Authors:** Xiaojian Gao, Xiaojun Zhang, Li Lin, Dongrui Yao, Jingjing Sun, Xuedi Du, Xiumei Li, Yue Zhang

**Affiliations:** 1 College of Animal Science and Technology, Yangzhou University, Yangzhou 225009, China; gaoxj336@163.com (X.G.); y0741810@126.com (J.S.); duxuedi@hotmail.com (X.D.); lixm0302@163.com (X.L.); 18861339319@163.com (Y.Z.); 2 College of Ocean, Huaihai Institute of Technology, Lianyungang 222005, China; 3 Department of Aquatic Animal Medicine, College of Fisheries, Huazhong Agricultural University, Wuhan 430070, China; 4 Institute of Botany Jiangsu Province and Chinese Academy Sciences, Nanjing 210014, China

**Keywords:** *Vibrio harveyi*, *Vibrio parahaemolyticus*, IgY, passive immunization, *Litopenaeus vannamei*

## Abstract

*Vibrio* spp. are major causes of mortality in white shrimp (*Litopenaeus vannamei*) which is lacking adaptive immunity. Passive immunization with a specific egg yolk antibody (IgY) is a potential method for the protection of shrimp against vibriosis. In this study, immune effects of the specific egg yolk powders (IgY) against both *V. harveyi* and *V. parahaemolyticus* on white shrimp were evaluated. The egg yolk powders against *V. harveyi* and *V. parahaemolyticus* for passive immunization of white shrimp were prepared, while a tube agglutination assay and an indirect enzyme-linked immunosorbent assay (ELISA) were used for detection of IgY titer. Anti-*Vibrio* egg yolk was encapsulated by β-cyclodextrin, which could keep the activity of the antibody in the gastrointestinal tract of shrimp. The results showed that the anti-*Vibrio* egg powders had an inhibiting effect on *V. harveyi* and *V. parahaemolyticus in vitro*. Lower mortality of infected zoeae, mysis, and postlarva was observed in groups fed with anti-*Vibrio* egg powders, compared with those fed with normal egg powders. The bacterial load in postlarva fed with specific egg powders in seeding ponds was significantly lower than those fed with normal egg powders in seeding ponds. These results show that passive immunization by oral administration with specific egg yolk powders (IgY) may provide a valuable protection of vibrio infections in white shrimp.

## 1. Introduction

*Vibrio* spp. is widely distributed in aquatic environments such as estuarine, coastal waters, and sediments. Vibriosis is one of the most prevalent infectious diseases in fish, shrimp, and shellfish species, particularly those cultivated in marine and estuarine environments [1]. Shrimps are important commercial aquaculture species in the world [2], but the industry of shrimp aquaculture has been threatened by *V. harveyi* and *V. parahaemolyticus* infections. *V. harveyi* could enter shrimp via the oral route, resulting in high mortality rates in shrimp farming [3]. Soto-Rodriguez *et al.* reported that the pathogenic *V. harveyi* was responsible for the “bright-red” syndrome in white shrimp [4]. *V. parahaemolyticus* is another important pathogen of white shrimp, which could cause acute hepatopancreatic necrosis disease [5]. In addition, *V. parahaemolyticus* is also a danger for human health, which is a leading cause of gastroenteritis in humans [6]. In our previous study, the strain of *V. harveyi* (KX1) caused mass mortality of mysis of *Fenneropenaeus chinensis* in a hatchery in Lianyungang, Jiangsu province [7]. In another study, the strain of *V. parahaemolyticus* (JGB080708-1) was isolated from diseased postlarva of *L. vannamei* with symptoms of sluggish swimming, black gill, and opaque muscle [8].

Antibiotics have been commonly used in the prevention and treatment of vibriosis. However, the abuse of antibiotics has led to food safety and the emerging of drug-resistance bacteria. [9]. It is general believed that there is no adaptive immunity in invertebrates [10]. Therefore, passive immunization is a promising way to protect invertebrates from infections. In recent years, the egg yolk antibody has attracted considerable attention because of its numerous advantages: it is stable, safe, economical, and easy to prepare at high concentrations [11,12,13]. IgY is successfully used in medical immune testing and the application on passive immunization in both animals and humans [14]. In aquatic animals, it has been shown that IgY has proven to be effective in protecting aquatic animals from a variety of pathogen infections, such as *Edwardsiella tarda*, *Yersinia ruckeri*, *Aeromonas hydrophila*, and *V. alginolyticus* [15]. However, there is no report about the effect of IgY on the protection of white shrimp against *V. harveyi* and *V. parahaemolyticus* infections.

In this report, we evaluated the protection of white shrimp against *V. harveyi* and *V. parahaemolyticus* infections using egg powders containing IgY. Oral administration with vibrio-specific IgY may be a promising strategy to fight against vibrio infections in white shrimp.

## 2. Results

### 2.1. Titer of Anti-Vibrio IgY in Egg Yolk

**Figure 1:**
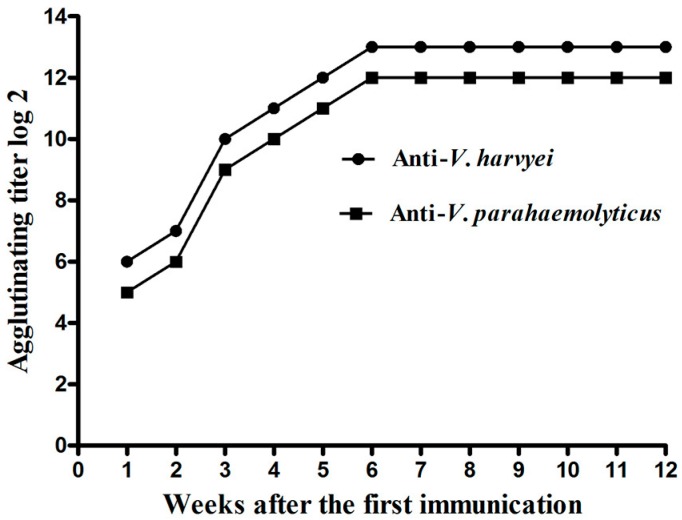
The titer of anti-*V. harveyi* and anti-*V. parahaemolyticus* IgY in the egg yolk detected by the tube agglutination test.

**Figure 2:**
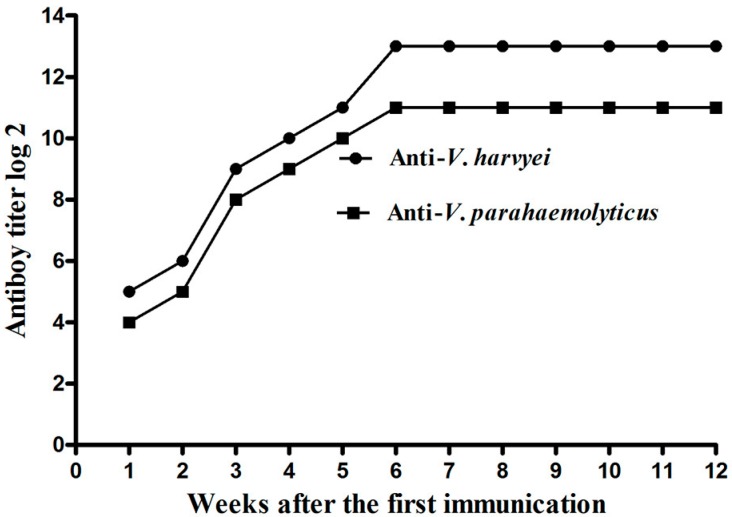
The titer of anti-*V. harveyi* and anti-*V. parahaemolyticus* IgY in the egg yolk detected by enzyme-linked immunosorbent assay (ELISA).

The antibody titer of IgY was determined with a tube agglutination test and an indirect enzyme-linked immunosorbent assay (ELISA) as described below. The results showed that injection with formalin-killed *V. harveyi*, and *V. parahaemolyticus* could effectively cause IgY accumulation in laying hens. As shown in Figure 1, the antibody titer in egg yolk increased slowly during the first 2 weeks after the first injection. The IgY titer showed a sharp increase at the end of the 3rd week after the first immunization. Six weeks after the first immunization, the titer of IgY against *V. harveyi* and *V. parahaemolyticus* reached a plateau and increased to 8192 and 4096, respectively. This high titer period lasted for more than 6 weeks. These changes of titer in egg yolk were also determined via ELISA, and the result was nearly consistent with agglutination titer (Figure 2). In contrast, the IgY titer for hens injected with a sterile saline solution was extremely low throughout the experiment. These results indicate that multiple immunizations could dramatically increase the antibody titer in egg yolk.

### 2.2. Titer of Anti-*Vibrio* IgY in Egg Yolk Powder

**Figure 3:**
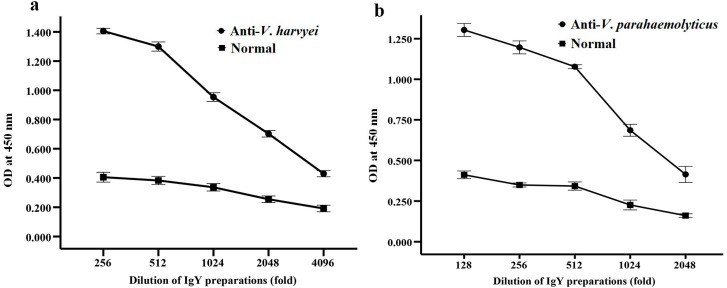
The titer of anti-*V. harveyi* and anti-*V. parahaemolyticus* IgY in the egg yolk powder by indirect ELISA. Data were presented as mean ± SD (*n* = 3). (**a**) The titer of the egg powder against *V. harveyi*; (**b**) The titer of the egg powder against *V. parahaemolyticus.*

The egg yolk powder was prepared by a spray-dried system as described below. The antibody titer in egg yolk powders was detected by indirect ELISA. The titers of specific egg yolk powder IgY against *V. harveyi* and *V. parahaemolyticus* were 4096 and 2048 (Figure 3), respectively. The antibody titer was expressed as the maximum dilution multiple in which the OD sample/OD negative exceeded 2.1. The titers of anti-*Vibrio* IgY in the egg yolk powder with antigen treatment are significant higher than those without antigen treatment (*p* < 0.05), indicating that the preparation of egg yolk powder IgY by coating with β-cyclodextrin followed by a spray-dried system was an effective method.

### 2.3. Inhibitory Effects of Anti-*Vibrio* IgY 

The bacteriostatic activity of the egg yolk powder was demonstrated as described below. As shown in Table 1, the maximum dilution of crude extraction of IgY against *V. harveyi* and *V. parahaemolyticus* were 1:16 and 1:8, respectively.

### 2.4. Absorption and Metabolism of IgY in White Shrimp

**Figure 4:**
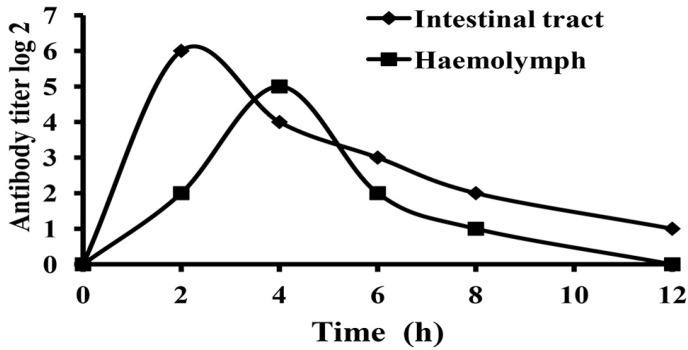
Different antibody levels in hemolymph and intestinal tract of *Litopenaeus vannamei* after oral administration the egg yolk powder by indirect ELISA.

As shown in Figure 4, the IgY antibody appeared in the intestinal tract; subsequently, it was detected in hemolymph. In addition, IgY could last for more than 12 h with activity in the intestinal tract of shrimp. The antibody level was monitored in the control group, and no IgY was detected. Therefore, the application of β-cyclodextrin microcapsules for coating egg yolk was an effective way to keep the activity of IgY in the gastrointestinal tract.

### 2.5. Effects of Dietary Anti-*Vibrio* Egg Yolk Powder on White Shrimp 

Two different kinds of feed (anti-*Vibrio* and normal egg yolk powders) were orally administrated to the white shrimp that were immersed with *V. harveyi* or *V. parahaemolyticus*, resulting in dramatic survival rates of white shrimp. The cumulative mortalities of specific egg yolk powders-treated zoeae, mysis, and postlarva of white shrimp were significantly lower than those of the untreated ones. As shown in Table 2, in test groups of bath challenged with *V. harveyi*, the cumulative mortalities of zoeae, mysis, and postlarva were 37.3%, 39.3%, and 38.0% at 48 h post of challenge, respectively, while the cumulative mortalities in control groups were 84.0%, 84.7%, and 88.0%, respectively, in test groups of bath challenged with *V. parahaemolyticus*, the cumulative mortalities of the zoeae, mysis, and postlarva were 40.0%, 41.4%, and 43.3%, respectively, while the mortalities in control groups were 86.7%, 84.0%, and 87.3%, respectively. These results indicated that the feeding of white shrimp with the specific egg yolk powders could protect them from *V. harveyi* and *V. parahaemolyticus* infection.

### 2.6. Effects of Anti-Vibrio Yolk Powder on the Bacterial Burden in Postlarva Cultured in Prawn Seeding Ponds

**Figure 5:**
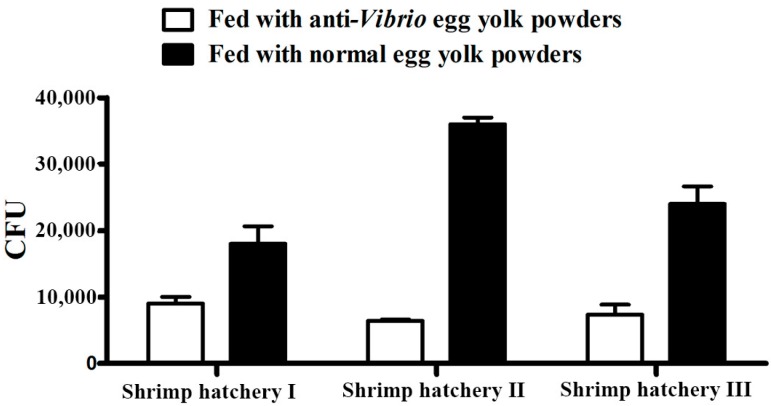
Bacterial burdens in postlarva from different shrimp hatcheries after oral anti-*Vibrio* yolk powder. Data are presented as CFU per g of postlarva. Bacterial burdens are significantly different with different treatments (*p* < 0.05). The results shown are the average of three independent experiments.

The number of colony forming units (CFUs) per g of white shrimp postlarva is shown in Figure 5. The bacterial burden in postlarve from different shrimp hatcheries fed with the specific egg yolk powders were 9 × 10^3^, 6.4 × 10^3^, and 8 × 10^3^ CFU per g, respectively, while the bacterial burden in those fed with normal egg yolk powder were 1.8 × 10^4^, 3.6 × 10^4^, and 2.4 × 10^4^ CFU per g, respectively.

## 3. Discussion

As a passive, stable, safe, inexpensive, and easily produced antibody, the specific IgY has drawn wide attention in passive immunization of aquatic animals against diseases in recent years [16,17]. Previous work has demonstrated that passive immunization using IgY is an effective way for protection of ayu (*Plecoglossus altivelis*) against *V. anguillarum*, crucian carp (*Carassius gibelio*) against *Aeromonas hydrophila*, Crayfish (*Procambius clarkiaii*), and *F. chinensis* against WSSV [11,18,19]. To obtain the egg yolk powders against both *V. harveyi* and *V. parahaemolyticus* used for the present study, laying hens were injected with formalin-killed *V. harveyi* and *V. parahaemolyticus,* emulsified with incomplete Freund’s adjuvant. The titer of IgY against both *V. harveyi* and *V. parahaemolyticus* increased to more than 4096 by the 6th week after primal immunization and retained high activity for the subsequent weeks. These data suggested that the formalin-killed whole cells of *V. harveyi* and *V. parahaemolyticus* were promising antigens for generating IgY in laying hen.

In this study, the biological activity of egg yolk powder containing IgY against both *V. harveyi* and *V. parahaemolyticus* was evaluated by detecting its titer and antibacterial activity. The highest titer for specific egg yolk powder containing IgY against *V. harveyi* and *V. parahaemolyticus* was 2048 and 4096, respectively. We also showed that IgY could inhibit the growth of *V. harveyi* and *V. parahaemolyticus* significantly *in vitro*, which agreed with the previous report [20]. The antibacterial mechanism of IgY is commonly considered as inhibition of adhesion of bacteria to the host cells [21]. The specific IgY may recognize and bind the particular components expressed on the bacterial surface, which are crucial factors for bacterial growth.

Our study demonstrated that the antibody level of the anti-*Vibrio* egg yolk powder IgY decreased within 12 h in the intestinal tract of white shrimp. How to keep the activity of IgY in the intestinal tract of shrimp is a key step for the effective application of IgY. It has been reported that the activity of anti-*Vibrio alginolyticus* IgY could be reduced or destroyed in the gastro-intestine of abalone [22]. Furthermore, the activity of anti-*Y. ruckeri* IgY decreased a lot after feeding for 2 h and was completely lost after feeding for 5 h in the stomach of rainbow trout [23]. Similarly, it was reported that IgY was sensitive to pepsin in pig, especially at pH < 4 [24]. Therefore, it would be useful to find an effective method to keep the activity of IgY during gastric passage. The applicability of chitosan-alginate microcapsules for coating IgY was reported to an effective way in pigs [25]. Additionally, hydrogels containing acrylamide and acrylic acid were used to coat IgY [26]. In this study, the applicability of β-cyclodextrin microcapsules for coating egg yolk was established. We showed the egg yolk powder containing IgY encapsulated with β-cyclodextrin could keep the IgY activity up to 12 h in the intestinal tract of white shrimp, which should enhance the effect of passive immunity of IgY.

Antibiotics have been commonly used in the prevention and treatment of bacterial disease in aquaculture. However, the misuse of antibiotics has led to problems with drug residues in fish products, increased bacterial resistance, and environmental pollution. In this study, immune effects of anti-*Vibrio* egg powders (IgY) against both *V. harveyi* and *V. parahaemolyticus* on white shrimp were evaluated, the results showed that survival rate of white shrimp zoeae, mysis, and postlarva was elevated after administered orally with the anti-*Vibrio* egg yolk powder when they were challenged with *V. harveyi* or *V. parahaemolyticus*. The survival rate of white shrimp zoeae, mysis, and postlarva elevated from 26%, 25.3%, 22% to 62.7%, 60.7%, and 62%, respectively, after being challenged with *V. harveyi.* Meanwhile, the survival rate of white shrimp zoeae, mysis, and postlarva elevated from 23.3%, 26%, 22% to 60%, 58.6%, and 56.7%, respectively, after being challenged with *V. parahaemolyticus*. In addition, oral administration of anti-*Vibrio* egg yolk powder could significantly decrease the bacterial burden of white shrimp postlarva in seeding ponds based on our experiments, which was consistent with previous reports [27]. These results indicated that anti-*Vibrio* egg yolk powder could be a potential biological agent in aquaculture. On the one hand, oral administration of anti-*Vibrio* egg yolk powder may enhance immunity and nutrition of animals. On the other hand, egg yolk powder containing an anti-*Vibrio* IgY antibody could inhibit adhesion of the *Vibrio* spp. to host cells.

In conclusion, our results indicated that laying hen injected with formalin-killed whole-cell of *V. harveyi* and *V. parahaemolyticus* can be promising antigens for generating high titer of IgY. Furthermore, β-cyclodextrin can tolerate the gastrointestinal digestion of white shrimp and will be a good candidate for the delivery of IgG in white shrimp. There may be great potential for commercial application to prevent vibriosis from using egg yolk powder containing IgY against *V. harveyi* and *V. parahaemolyticus* in white shrimp.

## 4. Materials and Methods

### 4.1. Preparation of Vaccine

The epidemic and virulent strain of *V. harveyi* (KX1) isolated from diseased mysis of *F. chinensis* [7] and *V. parahaemolyticus* (JGB080708-1) isolated from diseased *L. vannamei* [8], were used in this study. *V. harveyi* and *V. parahaemolyticus* were grown at 28 °C for 24 h in 2216E liquid medium, respectively. Cells were harvested using centrifugation at 8000× *g* for 10 min at 4 °C, washed with a sterile saline solution (0.9% *w*/*v* NaCl), and then re-suspended in the saline solution. The suspension was adjusted to a cell density of 10^9^ CFU/mL and was inactivated with 0.5% formaldehyde for 24 h at 28 °C. Complete killing of the two bacteria was confirmed by inoculating the treated samples on a TCBS agar plate and cultured overnight at 28 °C. Cells were washed twice with a sterile saline solution to remove the formaldehyde, and the two kinds of cells were mixed and re-suspended in the sterile saline solution. The suspensions of *V. harveyi* and *V. parahaemolyticus* were mixed with an appropriate leaching solution of astragals (1 mg/mL) as immunopotentiators and emulsified with an equal volume of incomplete Freund’s adjuvant. The inactivated vaccine was stored at approximately 4 °C before use.

### 4.2. Immunizations of Laying Hens

Thirty laying hens (120 days old) were kept in individual cages with ample feed and water throughout the course of the study and divided into control (5 hens) and test groups (25 hens). In each test group, each hen was immunized with a 0.5-mL dose of vaccine into 2 sites at the neck subcuticle. After the first immunization, three additional booster immunizations were administered at 1-week intervals. The second, third, and fourth injections were given at the same route with the first injection but at a double dose. The control group was injected with a sterile saline solution with the same route in test group. The eggs from the immunized and control hens were collected and stored at 4 °C.

### 4.3. Detection of IgY in Yolk by Tube Agglutination Test

The antibody titer of the egg yolk was determined with a tube agglutination test [28]. Briefly, the test was conducted in 15 sterile test tubes of 100 × 10 mm. The egg yolk was subjected to serially two-fold dilution in a sterile saline solution, ranging from 1:2 to 1:32,768. An amount of 0.2 mL of *V. harveyi* or *V. parahaemolyticus* (10^9^ CFU/mL) was added into each tube. The tubes were then shaken and incubated at room temperature (25 °C) for 24 h. Results of the reaction were observed visually and an umbrella-like white clump at the bottom of the tube indicated a positive reaction. The non-reactor tubes did not show any clump.

### 4.4. Detection of IgY in Yolk by ELISA

The antibody titer of the egg yolk was determined via indirect enzyme-linked immunosorbent assay (ELISA) [29]. Briefly, the egg yolk was separated and diluted with 4 volumes of sterile double distilled water that was acidified with concentrated HCl to obtain a final pH of 5.0. The water-soluble fraction was then subjected to serially two-fold dilution. 96-well ELISA plates (Costar, New York, NY, USA) were coated with 100 μL of *V. harveyi* or *V. parahaemolyticus* (10^9^ CFU/mL) in bicarbonate buffer (0.05 M, pH 9.6) and stored at 37 °C for 2 h. The plates were washed three times with phosphate buffer saline (PBS) containing 0.05% Tween-20 (PBST); subsequently, each well was blocked with 100 μL of PBS containing 1% bovine serum albumin (PBSA) at 37 °C for 2 h. After three rinses with PBST, 100 μL of the serial dilutions of IgY sample was added to each well and the plate was then incubated at 37 °C for 2 h. The plate was washed again, and each well was treated with 100 μL of rabbit anti-chicken IgG conjugated with horse radish peroxidase (Auragene Bioscience, Co., Ltd., Changsha, China. 1:10,000) and incubated at 37 °C for 2 h. After washing 5 times, 100 mL of 3,3′,5,5′-Tetramethylbenzidine (TMB) substrate solution (Sigma, St. Louis, MO, USA) was added to each well. The plate was incubated at 37 °C for 15 min, and 50 μL of 2 M H_2_SO_4_ was then added to each well to stop the reaction. The color developed in the wells was measured at 450 nm with a plate reader (model Synergy HT, Bio-Tek, Winooski, VT, USA). The antibody titer was expressed as the maximum dilution multiple in which the OD sample/OD negative exceeded 2.1.

### 4.5. Preparation of Egg Yolk Powders Containing Anti-Vibiro IgY

Four weeks after the primary immunization, the eggs were collected and used to prepare egg powders containing anti-*Vibrio* IgY. Briefly, the egg yolk was diluted twice in a sterile saline solution and dissolved in 5% β-cyclodextrin (*w*/*v*, final concentration). The β-cyclodextrin coated egg yolk was spray-dried at 150 °C. The antibody titer of the egg yolk powder was determined via indirect ELISA as described above.

### 4.6. Bacterial Inhibiting Assay

We investigated whether the anti-*Vibrio* IgY could inhibit the *V. harveyi* and *V. parahaemolyticusin* in LB broth medium. The same strains of *V. harveyi* and *V. parahaemolyticus* which were used as the antigen for immunizing chickens were cultured in nutrient broth, respectively. The suspensions were adjusted to a cell density of 10^5^ CFU/mL by the sterile saline solution. 100 g of egg yolk powder was diluted 4 times in double distilled water that was acidified with concentrated HCl to obtain a final pH of 5.0. After incubation at 4 °C for 5 h, the solution was centrifuged at 8000× *g* (4 °C, 30 min), and the supernatant was clarified by filtration through a 0.45-μm filter before adding ammonium sulfate (up to 50%). After incubation at 4 °C for 18 h, the solution was centrifuged at 8000× *g* (4 °C, 30 min), and the supernatant was discarded. The pellet was re-suspended with 100 mL of PBS and dialyzed against PBS. The bacteria in the crude extraction of IgY were eliminated by filtration through a 0.22-μm filter. The crude extraction of IgY was then subjected to serially two-fold dilution by sterile LB broth medium. The bacterial inhibiting test was conducted in 10 sterile test tubes. One milliliter of the serial dilutions of crude extraction of IgY was added into each tube, and 10 μL of bacterial solution was added and incubated at 28 °C for 16 h. The suitable specific antibody concentration was defined as the lowest concentration at which no microbial growth could be observed.

### 4.7. Kinetic of IgY in White Shrimp

A total of 50 white shrimp (4–5 cm length) were obtained from a farm located in Lianyungang city, Jiangsu province, P.R. China and acclimated in a 20-L tank for 3 days. These shrimps were divided into test and control groups. The shrimps in control or test groups were fed with a basal diet containing 15% of anti-*Vibrio* or normal egg yolk powder, respectively, before they were starved for 24 h. Hemolymph and intestinal tract of shrimps were sampled after feeding the diets for 2, 4, 6, 8, and 12 h, respectively. Then, the hepatopancreas and intestinal tract were slightly washed with a sterile saline solution, and each sample of intestinal tract was homogenized in 0.4 mL of sterile PBS (pH 7.4). Subsequently, these samples were centrifuged at 8000× *g* (4 °C, 30 min), and the antibody level of supernatant was assayed by indirect ELISA as described above.

### 4.8. Challenge Experiment

The protective effect of anti-*Vibrio* egg yolk powder on white shrimp against the bacterial infections was assessed by bath infection with *V. harveyi* and *V. parahaemolyticus* (10^6^ CFU/mL), severally. Briefly, a number of zoeae, mysis, and postlarva of white shrimp were obtained from a farm located in Lianyungang city and were acclimated for a week in a 20-L tank, respectively. These shrimps were divided into test groups and control groups. A total of 150 of each zoeae, mysis, and postlarva were kept in a 2-L beaker, respectively. Groups of zoeae, mysis, and postlarva were fed with 4 g/mL of a basal diet containing anti-*Vibrio* or normal egg yolk powders per day and had lasted for a week. At Day 8, groups of zoeae, mysis, and postlarva were exposed to 1.8 × 10^6^ CFU/mL *V. harveyi* and *V. parahaemolyticus*, severally. Morbidity of zoeae, mysis, and postlarva was monitored for 48 h after challenge, and the results were recorded every 6 h.

### 4.9. Effects of Anti-Vibrio Yolk Powder on the Bacterial Burden in Postlarva of White Shrimp

Effects of anti-*Vibrio* egg yolk powder on the bacterial burden were evaluated in three white shrimp hatchery farms located in Ganyu county, Jiangsu province, P. R. China. The experiments were conducted in prawn seeding ponds (8 × 6 m^2^) including three test and three control groups in each shrimp hatchery, the water level of each pond was kept at a depth of approximately 60 cm, and the density of the postlarva was 80,000/m^3^. The postlarva in control or test groups were fed with a basal diet containing 15% of anti-*Vibrio* or normal egg yolk powder. The bacterial burden in postlarva was detected after 15 days. 1 mg of postlarva was homogenized in 1 ml of sterile PBS (pH 7.4). Homogenates were serially diluted in a sterile PBS (pH 7.4) and plated onto separate thiosulfate citrate bile salts sucrose (TCBS) agar plates. After incubation for 18 h at 28 °C, bacterial colonies in the plates were counted separately for each sample.

### 4.10. Statistical Analysis

SPSS 16.0 statistical software was used to analyze data, and data were presented as means ± standard deviation (SD). Statistical significance was assessed by using a one-way ANOVA and Tukey test, and *p* < 0.05 was considered as significant difference.

## Figures and Tables

**Table 1:** The inhibitory effect of specific IgY on *V. harveyi* and *V. parahaemolyticus.*

Strain	Dilution of Crude Extraction of IgY (Fold)								
	1:2	1:4	1:8	1:16	1:32	1:64	1:128	1:256	1:512
*V. harveyi*	−	−	−	−	+	+	+	+	+
*V. parahaemolyticus*	−	−	−	+	+	+	+	+	+

“+” microbial growth; “−“ no microbial growth.

**Table 2:** Effects of dietary specific egg yolk powder on the survival rate of zoeae, mysis, and postlarva.

Bacteria	Groups	Feed	Dead Larva After Challenge (h)					Total Deaths	Mortality (%)
			6	12	18	24	48		
*V. harveyi*	Zoea	Specific IgY	6	10	12	13	15	56	37.3
		Normal IgY	24	28	21	29	24	126	84.0
	Mysis	Specific IgY	5	10	13	15	16	59	39.3
		Normal IgY	19	28	30	23	27	127	84.7
	Postlarva	Specific IgY	8	11	13	12	13	53	38.0
		Normal IgY	16	24	28	29	35	132	88.0
*V. parahaemolyticus*	Zoea	Specific IgY	4	12	15	17	12	60	40.0
		Normal IgY	34	30	23	19	24	130	86.7
	Mysis	Specific IgY	3	12	16	18	13	62	41.4
		Normal IgY	21	28	32	23	22	126	84.0
	Postlarva	Specific IgY	8	18	13	11	15	65	43.3
		Normal IgY	15	23	29	26	38	131	87.3

## References

[1] Lun J.S., Xia C.Y., Yuan C.F., Zhang Y.L., Zhong M.Q., Huang T.W., Hu Z. (2014). The outer membrane protein, LamB (maltoporin), is a versatile vaccine candidate among the *Vibrio* species. Vaccine.

[2] Sun R., Qiu L.M., Yue F., Wang L.L., Liu R., Zhou Z., Zhang H., Song L.S. (2013). Hemocytic immune responses triggered by CpG ODNs in shrimp *Litopenaeus vannamei*. Fish Shellfish Immunol..

[3] Rungrassamee W., Klanchui A., Maibunkaew S., Karoonuthaisiri N. (2016). Bacterial dynamics in intestines of the black tiger shrimp and the Pacific white shrimp during *Vibrio harveyi* exposure. J. Invertebr. Pathol..

[4] Soto-Rodriguez S.A., Gomez-Gil B., Lozano R., Rio-Rodríguez R. (2012). Virulence of *Vibrio harveyi* responsible for the “Bright-red” Syndrome in the Pacific white shrimp *Litopenaeus vannamei*. J. Invertebr. Pathol..

[5] Tang K.F.J., Lightner D.V. (2014). Homologues of insecticidal toxin complex genes within a genomic island in the marine bacterium *Vibrio parahaemolyticus*. FEMS Microbiol. Lett..

[6] Lomelí-Ortega C.O., Martínez-Díaz S.F. (2014). Phage therapy against *Vibrio parahaemolyticus* infection in thewhiteleg shrimp (*Litopenaeus vannamei*) larvae. Aquaculture.

[7] Zhang X.J., Bi K.R., Yan B.L., Chen L., Bai X.S., Qin L. (2014). Identification and virulence genes detection of pathogenic *Vibrio harveyi* isolated from mysis of *Fenneropenaeus chinensis* L.. Prog. Fish. Sci..

[8] Zhang X.J., Chen C.Z., Yan B.L., Fang H., Qin G.M., Xu J. (2009). Phenotypic and molecular characterization of pathogenic *Vibrio parahaemolyticus* isolated from *Penaeus vannamei*. Oceanol. Limnol. Sin..

[9] Dang H.Y., Zhao J.Y., Song L.S., Chen M.N., Chang Y.Q. (2009). Molecular characterizations of chloramphenicol-and oxytetracycline-resistant bacteria and resistance genes in mariculture waters of China. Mar. Pollut. Bull..

[10] Zhang Y., Qiu L.M., Song L.S., Zhang H., Zhao J.M., Wang L.L., Yu Y.D., Li C.H., Li F.M., Xing K.Z. (2009). Cloning and characterization of a novel C-type lectin gene from shrimp *Litopenaeus vannamei*. Fish Shellfish Immunol..

[11] Andrade F.G., Eto S.F., Ferraro A.C.N.S., Marioto D.T.G., Vieira N.J., Cheirubim A.P., Ramos S.P., Venâncio E.J. (2013). The production and characterization of anti-bothropic and anti-crotalic IgY antibodies in laying hens: A long term experiment. Toxicon.

[12] Amaral J.A., Franco M.T., Zapata-Quintanilla L., Carbonare S.B. (2008). *In vitro* reactivity and growth inhibition of EPEC serotype O111 and STEC serotypes O111 and O157 by homologous and heterologous chicken egg yolk antibody. Vet. Res. Commun..

[13] Lu Y.N., Liu J.J., Jin L.J., Li X.Y., Zhen Y.H., Xue H.Y., You J.S., Xu Y.P. (2008). Passive protection of shrimp against white spot syndrome virus (WSSV) using specific antibody from egg yolk of chickens immunized with inactivated virus or a WSSV-DNA vaccine. Fish Shellfish Immunol..

[14] Spillner E., Braren I., Greunke K., Seismann H., Blank S., Plessis D. (2012). Avian IgY antibodies and their recombinant equivalents in research, diagnostics and therapy. Biologicals.

[15] Gan H.J., He H.W., Sato A., Hatta H., Nakao M., Somamoto T. (2015). Ulcer disease prophylaxis in koi carp by bath immersion with chicken egg yolk containing anti-*Aeromonas salmonicida* IgY. Res. Vet. Sci..

[16] Silva W.D., Tambourgi D.V. (2010). IgY: A promising antibody for use in immunodiagnostic and in immunotherapy. Vet. Immunol. Immunopathol..

[17] Xu Y.P., Li X.Y., Jin L.J., Zhen Y.H., Lu Y.N., Li S.Y., You J.S., Wang L.H. (2011). Application of chicken egg yolk immunoglobulins in the control of terrestrial and aquatic animal diseases: A review. Biotechnol. Adv..

[18] Jin L.J., Li X.Y., Zou D.L., Li S.Y., Song W.Q., Xu Y.P. (2013). Protection of crucian carp (*Carassius auratus Gibelio*) against septicaemia caused by *Aeromonas hydrophila using* specific egg yolk immunoglobulins. Aquac. Res..

[19] Lu Y.N., Liu J.J., Jin L.J., Li X.Y., Zhen Y.H., Xue H.Y., Lin Q.Y., Xu Y.P. (2009). Passive immunization of crayfish (*Procambius clarkiaii*) with chicken egg yolk immunoglobulin (IgY) against white spot syndrome virus (WSSV). Appl. Biochem. Biotechnol..

[20] Li X.Y., Liu H., Xu Y.P., Xu F.X., Wang L.H., You J.S., Li S.Y., Jin L.J. (2012). Chicken egg yolk antibody (IgY) controls *Solobacterium moorei* under *in vitro* and *in vivo* conditions. Appl. Biochem. Biotechnol..

[21] Ibrahim E.M., Rahman A.K.M.S., Isoda R., Umeda K., Sa N.V., Kodamaa Y. (2008). *In vitro* and *in vivo* effectiveness of egg yolk antibody against *Candida albicans* (anti-CA IgY). Vaccine.

[22] Wu C.J., Wang H., Chan Y.L., Li T.L. (2011). Passive immune-protection of small abalone against *Vibrio alginolyticus* infection by anti-*Vibrio* IgY-encapsulated feed. Fish Shellfish Immunol..

[23] Lee S.B., Mine Y., Stevenson R.M. (1999). Effects of hen egg yolk immunoglobulin in passive protection of rainbow trout against *Yersinia ruckeri*. J. Agric. Food Chem..

[24] Fan J.H., Zuo Y.Z., Li T.Q., Zhang X.B. (2009). Preparation and physicochemical property of chicken yolk immunoglobulin (IgY) against porcine transmissible gastroenteritis virus (TGEV). Front. Agric. China.

[25] Li X.Y., Jin L.J., Uzonna J.E., Li S.Y., Liu J.J., Li H.Q., Lu Y.N., Zhen Y.H., Xu Y.P. (2009). Chitosan-alginate microcapsules for oral delivery of egg yolk immunoglobulin (IgY): *In vivo* evaluation in a pig model of enteric colibacillosis. Vet. Immunol. Immunopathol..

[26] Bellingeri R.V., Picco N.Y., Alustiza F.E., Canova J.V., Molina M.A., Acevedo D.F., Barbero C., Vivas A.B. (2015). pH-responsive hydrogels to protect IgY from gastric conditions: *In vitro* evaluation. J. Food Sci. Technol..

[27] Li X.Y., Jing K.L., Wang X.T., Li Y., Zhang M.X., Li Z., Xu L., Wang L.L., Xu Y.P. (2016). Protective effects of chicken egg yolk antibody (IgY) against experimental *Vibrio splendidus* infection in the sea cucumber (*Apostichopus japonicus*). Fish Shellfish Immunol..

[28] Banerjee D.P., Sharma S.K., Gautam O.P., Sarup S. (1978). The use of spleen antigen in the tube agglutination test for diagnosis of anaplasmosis in cattle. Trop. Anim. Health Prod..

[29] Li C.H., Lu X.J., Li D.F., Chen J. (2014). Passive protective effect of chicken egg yolk immunoglobulins against experimental *Vibrio anguillarum* infection in ayu (*Plecoglossus altivelis*). Fish Shellfish Immunol..

